# Genome-Wide Association Studies: Progress in Identifying Genetic Biomarkers in Common, Complex Diseases

**Published:** 2007-08-08

**Authors:** Stephen F. Kingsmore, Ingrid E. Lindquist, Joann Mudge, William D. Beavis

**Affiliations:** National Center for Genome Resources, Santa Fe, NM 87505, U.S.A

**Keywords:** Genome-wide association studies, Complex diseases, Complex traits, Genetic biomarkers, Population genetics

## Abstract

Novel, comprehensive approaches for biomarker discovery and validation are urgently needed. One particular area of methodologic need is for discovery of novel genetic biomarkers in complex diseases and traits. Here, we review recent successes in the use of genome wide association (GWA) approaches to identify genetic biomarkers in common human diseases and traits. Such studies are yielding initial insights into the allelic architecture of complex traits. In general, it appears that complex diseases are associated with many common polymorphisms, implying profound genetic heterogeneity between affected individuals.

## Introduction

Genetic biomarkers are uniquely important since they are unambiguously associated with causality of diseases, traits or phenotypes. In common with other types of biomarkers, they are useful for diagnosis, patient stratification and prognostic or therapeutic categorization. Distinctively, however, they are frequently useful for provision of novel insights into disease pathogenesis and, thereby, novel therapeutic targets and strategies. Furthermore, inherited genetic biomarkers are present at birth, enabling institution of timely preventative or ameliorative measures.

During the past 25 years, genetic linkage studies have been exceptionally effective in identifying genetic biomarkers in Mendelian (simple or single gene) disorders. These studies have identified causal gene variants in more than 1300 Mendelian diseases (Botstein and Risch, 2003). Most common diseases, traits and phenotypes, however, do not exhibit Mendelian patterns of inheritance, but rather exhibit complex, multifactorial expression and inheritance. None-the-less, linkage based methods, especially the transmission disequilibrium test (Spielman et al. 1993) were developed and applied to a number of complex traits in the 1990s. These linkage studies met with little success in the identification of the allelic determinants of these common, complex disorders or traits (Freimer and Sabatti, 2004). In particular, there has been a lack of replication among studies, whereby an initial study will identify a genotype with large estimated genetic effects (relative risk) but subsequent studies will not corroborate the results (Lander and Kruglyak, 1995; Göring et al. 2001). In part, this reflects the dependence of linkage studies on unusually informative pedigrees (with multiple affected and unaffected individuals), which induce a bias toward rare, semi-Mendelian disease subsets in sub-populations. Tremendous excitement, therefore, has met recent reports of successful identification of genetic biomarkers in complex traits using an approach that does not have this limitation—genome wide association (GWA) studies.

Genome wide association studies are predicated on two observations:

1. The brief history of most human populations precludes sufficient number of generations (or meioses) to create recombination events (or mutations) between closely linked markers; and
2. Suppression of meiotic recombination (coldspots) occurs very frequently in mammalian genomes.

Thus, approximately 80% of the human genome is comprised of ~10 kilobase regions (haplotype blocks, HB) that do not show recombination in human populations (International HapMap Consortium, 2005). Genetic variants within HB are in linkage disequilibrium (LD). This phenomenon enables much of the recombination history in a population to be ascertained by genotyping a large set of randomly spaced tags throughout the genome, especially if these tags are located within HB. During the last ten years, more than ten million single nucleotide polymorphisms (SNPs) have been aggregated in a public database (Sherry et al. 2001). Furthermore, the International HapMap project has genotyped over three million of these SNPs that are common (occur with a minor allele frequency of >5%) in human populations and have assembled these genotypes computationally into a genome-wide map of SNP-tagged HB (International HapMap Consortium, 2005). These resources, together with array technologies for massively parallel SNP genotyping, have made GWA studies feasible. Further, the well established epidemiological case-control experimental design is directly applicable to GWA studies.

Initially, association genetic studies (focused on candidate genetic loci) also exhibited a lack of replication among studies (Ionnidis et al. 2001; Hirschhorn et al. 2002). The potential reasons for inconsistent results consist of unobserved, confounding biological sources of heterogeneity including inconsistent or poorly defined measurements of the phenotype, heterogeneous genetic sources for the phenotype, population stratification, population-specific LD, heterogeneous genetic and epigenetic backgrounds, or heterogeneous environmental influences. In addition to these biological explanations, there are statistical reasons for inconsistent results including failure to control the rate of false discoveries, lack of power, model misspecification and heterogeneous bias in estimated effects among studies (Cardon and Bell, 2001; Cardon and Palmer, 2003; Redden and Allison, 2003; Sillanpää and Auranen, 2004). Among these, the most likely source of non-replication is the lack of power due to the limited number of individuals genotyped and phenotyped in these experiments (Risch, 2000; Lohmueller et al. 2003). In the past year, however, a significant number of studies have been published that have used SNP genotyping arrays in large cohorts of individuals resulting in replicated associations between individual SNP-tagged HB and common, complex traits, phenotypes or diseases. In common with other biomarker development approaches, GWA study designs entail discovery, validation and replication stages (Kingsmore, 2006). This design is critical for detection of meaningful, hypothesis-generating genotype-phenotype associations given the large number of comparisons involved, prior probability estimates of association, sample sizes, resampling procedures, and statistical significance thresholds.

## Results of Initial GWA Studies

The first GWA study, published in 2002, examined myocardial infarction (Ozaki et al. 2002). The discovery phase comprised genotyping of 65,671 informative, coding domain SNPs (cSNPs) in 752 cases and control individuals (Table 1). Gene-tagging SNPs are more informative than random SNPs, since the vast majority of true-positive associations will be with genes (Botstein and Risch, 2003). The validation phase featured 26 SNPs and 2137 individuals and confirmed an association with a 50 kb HB containing the lymphotoxin-α (LTA), nuclear factor of kappa light polypeptide gene enhancer in B cells, inhibitor-like 1 (NFKBIL1) and HLA-B associated transcript 1 (BAT1) genes (Table 2). Replication studies have subsequently been undertaken, some of which have confirmed association of this region to myocardial infarction-related phenotypes, and, in particular, to a nonsynonymous SNP (nsSNP) in LTA (Yamada et al. 2004; Laxton et al. 2005; Mizuno et al. 2006; Clarke et al. 2006; Kimura et al. 2007; Sedlacek et al. 2007; Koch et al. 2007). As with most GWA studies, the association of LTA with myocardial infarction was an unexpected finding and suggests novel therapeutic approaches.

A second, pioneering GWA study examined age-related macular degeneration (AMD) (Klein et al. 2005, Table 1). The discovery phase genotyped 105,980 SNPs in only 146 cases and control individuals. SNPs in the complement factor H (CFH) gene, including an nsSNP, showed significant association with AMD. A validation phase was not performed, but numerous subsequent studies have replicated associations of CFH variants with protection and predisposition to AMD (Zareparsi et al. 2005; Hageman et al. 2005; Souied et al. 2005; Magnusson et al. 2006). Of all complex traits examined by GWA to date, AMD is unique in that a single, novel HB explained 61% of the genetic variance, conferring an odds ratio (OR) of 7.4 (Table 2). To put this in perspective, this OR is of similar magnitude to the classic associations of HLA-B27 with anterior uveitis/ankylosing spondylitis and HLA alleles with type 1 diabetes mellitus. Complement pathway dysregulation was a novel, unexpected association with AMD. Excitingly, this defect is likely to be therapeutically tractable.

During 2006, considerable technical and cost-effectiveness challenges were overcome, resulting in broad adoption and numerous GWA study publications.

Five large GWA studies have examined Crohn’s disease and ulcerative colitis, the two most common types of inflammatory bowel disease (IBD) (Table 1). Three of the studies used microarrays featuring ~300,000 random SNPs (Duerr et al. 2006; Rioux et al. 2007; Libioulle et al. 2007), while the fourth study used custom chips featuring ~16,000 non-synonymous SNPs (Hampe et al. 2007). The behemoth fifth study used 469,557 random SNPs in 14,000 individuals with seven common diseases but lacked replication (WTCCC 2007). Of 14 novel HB associations, 10 were unique to a single study. Three HB were concordant in four of the five studies (representing the genes CARD15, IL23R and ATG16L1). One HB was identified in two of the five studies (PTGER4). Estimated genetic effects, i.e. relative risks, of associated loci were small; the cumulative odds ratio associated with 28 risk alleles was only 1.45. Several studies sought evidence for epistatic interactions between IBD-associated HB. Two studies found suggestive evidence for epistasis involving two different pairs of HB. As yet, IBD candidate genes do not appear to be coalescing into biologic networks or pathways.

Six large GWA studies have examined type II (or adult onset) diabetes mellitus (T2DM), providing the best example to date of capabilities and limitations of GWA studies (Diabetes Genetics Initiative, Broad Inst. et al. 2007; Scott et al. 2007; Sladek et al. 2007; Steinthorsdottir et al. 2007; Zeggini et al. 2007; WTCCC 2007; Table 1). The discovery phase of these studies comprised genotyping of 313,179–469,557 SNPs in 2,335–16,179 individuals. Several studies sought association both with SNPs and with haplotype blocks in the discovery phase. Many of these studies were very large. Case-control and family-based association statistics were employed in most of the studies. Two employed over 9,000 individuals in the validation phase while one lacked replication.

Concordance of associated genes between T2DM GWA studies was striking; of 10 novel HB associations, only two were unique to a single study. Discordance of associations partly reflected different coverage of specific HB by the two microarray platforms used for genotyping. In common with IBD, T2DM-associated HB exhibited small estimated genotypic effect sizes. In contrast to IBD, however, many of the T2DM-associated candidate genes coalesced into biologic pathways, such as pancreatic islet beta cell function, including insulin biosynthesis.

Three studies performed initial modeling of how loci combine to affect susceptibility to T2DM. One study found suggestive evidence for epistatic interactions between two HB. Otherwise, it appeared that T2DM fits a polygenic threshold model with additive/multiplicative effects of individual loci.

As anticipated, allele frequencies showed considerable variation between ethnic and racial groups. Somewhat surprising, however, was the incidence of conservation of T2DM and IBD associated risk alleles between independent populations.

Two studies extended GWA analysis of T2DM to endophenotypes related to serum triglycerides and obesity (Diabetes Genetics Initiative, Broad Inst. et al. 2007; Frayling et al. 2007; Table 1). For example, one gene associated with T2DM, fat mass and obesity associated gene (FTO) (Scott et al. 2007), also showed an association with obesity-associated quantitative traits in an independent study (Frayling et al. 2007). Frayling et al. examined the association of the FTO variant with body mass index (BMI) in 13 cohorts with 38,759 participants. The association of FTO SNPs with obesity has been independently confirmed in 8000 individuals (Dina et al. 2007).

Cancers are fascinating genetic diseases as they feature the combined effects of germline risk alleles and multiple somatic mutations. Three GWA studies sought inherited haplotype block associations with breast cancer (Easton et al. 2007; Stacey et al. 2007; Hunter et al. 2007). The discovery phase of these studies comprised genotyping of 227,876–528,173 SNPs in 2,287–13,163 individuals. Validation phases varied in size from 3,848–44,438 individuals. Associations were identified in SNPs in FGFR2 (two studies), TNRC9 (two studies), at haplotype blocks rs13387042 and rs3817198 (which do not contain known genes, one study each), MAP3K1 (one study) and LSP1 (one study).

Two GWA studies sought association signals in prostate cancer (Gudmundsson et al. 2007; Yeager et al. 2007). The discovery phase of these studies comprised genotyping of 316,515–550,00 SNPs in 2,339–4,517 individuals. Validation phases varied in size from 3,655–6,266 individuals. An association with a HB at 8q24 that had previously been identified by linkage analysis (Amundadottir et al. 2006) was seen in both studies. In addition, both studies identified a second 8q24 HB, ~300 kb upstream from the first. As yet, the functional basis of these associations is unclear. While individual 8q24 variants showed modest estimated genetic effects, the cumulative effect of several variants fits a multiplicative model that conferred a population attributable risk (PAR), the expected reduction in prostate cancer incidence if the risk alleles did not exist in the population, of up to 68% (Haiman et al. 2007).

The applicability of GWA studies to complex traits has also been demonstrated. One study undertook GWA with numerous quantitative and categorical memory-associated endophenotypes (Papassotiropoulos et al. 2006). Despite the small size of the discovery cohort (341 individuals), associations with one HB (in the KIBRA gene) were replicated in two validation cohorts (totaling 680 individuals). A notable strength of this study was that associations were sought with multiple types of endophenotypes (performance in seven memory-associated tests and functional magnetic resonance image-based measures of the hippocampus during three memory-associated tests).

In addition to the identification of novel HB associations, GWA studies have confirmed several associations of susceptibility genes that were previously established by linkage analysis in large pedigrees. For example, a GWA study of 502,627 SNPs in 1086 cases of late-onset Alzheimer’s disease and controls verified the well established APOE susceptibility gene (Coon et al. 2007).

A remaining problem with large GWA studies is genotyping cost. One innovative study provided evidence that sample pooling strategies may be effective. In a GWA study of bipolar disorder, investigators created 39 pools, each containing equimolar amounts of DNA from 42–80 individuals (Baum et al. 2007). These pools represented a discovery and replication cohort. Pools were individually genotyped for 555,235 SNPs and normalized allele frequencies were inferred from intensity data. Replicates were assayed for each pool. 37 SNPs showing allele frequency differences in both cohorts were individually genotyped and 76% retained significant associations.

## Conclusions

During the past year, the utility of GWA studies for identification of novel genomic associations with complex disorders has unambiguously been established. In general these studies have employed large case-control cohorts featuring both familial and sporadic cases, categorical trait definitions and up to half a million commonly polymorphic SNPs. Excitingly, these studies are starting to provide empiric data to resolve decades of debate about the genetic architecture of complex traits. To date, with the exception of CFH in AMD, the estimated genetic effects of replicated associations have been uniformly and surprisingly small (Table 2). Also surprising is the high frequency of many risk alleles, albeit this may reflect an artifact induced by use of genotyping arrays that primarily feature common polymorphisms (Table 3). Informed by studies to date, the picture of the genetic architecture of complex traits that is emerging is immense polygenicity and individual genetic heterogeneity. In general, the data fit additive, threshold models. In a handful of informative studies, little evidence for epistasis has been observed. If confirmed, an implication will be that genetic diagnostics are still a long way off and will certainly not result in the deterministic prognostications portrayed in the 1997 movie Gattaca.

Encouragingly, most associated haplotype blocks are small enough to feature a single gene (Table 3). In large measure, this reflects the use of more outbred populations in fine-mapping validation phases. Furthermore, many HB contain a single, unequivocally functional variant. The distribution of variants does not yet show much difference from causal variants identified in Mendelian disorders. Thus, nsSNPs are relatively common and rSNPs are uncommon, a controversial point (Botstein and Risch, 2003; Knight, 2005; Thomas and Kejariwal, 2004). The vast majority of associated genes identified to date were not candidate genes previously, continuing the marvelous tendency of comprehensive, genetic-driven studies to be hypothesis-informing. It is refreshing to see associations with genes that had never previously been considered in a disease or trait. As yet, the confluence of associated genes into biologic networks and pathways has been disappointing. Surprisingly, there appears to be significant conservation of variant associations between human populations (albeit in the setting of frequently different allele frequencies). The emerging, principal benefit of GWA studies may therefore be elucidation of molecular mechanisms underpinning poorly understood diseases and traits.

In the few informative studies reported to date, endophenotypes have been highly instructive in dissecting the network or pathway perturbed by an individual variant to impact a complex trait. It is particularly exciting to see the application of multimode endophenotypes, such as combinations of psychological testing, brain imaging and gene expression in one study (Papassotiropoulos et al. 2006). The very large cohorts needed to discover and validate variants with small effect sizes preclude the collection of rich, accurate metadata. It is likely that future studies will utilize much greater stratification of traits than the phenotypically crude studies reported to date. Recent GWA studies of breast cancer provide a good example of the added genetic complexity that can be revealed by trait stratification (Easton et al. 2007; Hunter et al. 2007; Stacey et al. 2007). In addition, following replication of associations with categorical traits, it is anticipated that targeted genotypic examination of many endophenotypes will be highly instructive in the dissection of disease pathogenesis.

## Future Developments and Implications

Several trends observed in GWA studies to date are anticipated to continue. One million SNP chips are about to be launched and genotype accuracies have improved. Cohort sizes are increasing. Combinations of genotype-based and haplotype-based associations are becoming more prevalent. Experimental designs and statistical methods are becoming more uniform, enabling more meaningful meta-analyses. In particular, the emergence of adaptive designs and the use of Bayesian inferential methods produce probabilistic synthesis from combined analysis (Barry, 2005). Importantly, this will provide an intuitive framework for combining information from multiple studies resulting in more effective utilization of patient information from translational research—especially for detection and validation of weak associations.

As noted above, phenotypes remain crude and the use of endophenotypes or component phenotypes is anticipated to increase significantly. In particular, biomarker phenotypes are anticipated to become widely used. These are likely to include gene expression, proteomic, metabolomic and imaging biomarkers. As determinants of complex traits are identified, genetic stratification of cases and controls will be possible, reducing the genetic complexity of the trait and enabling identification of additional association signals. An area of substantial interest for the pharmaceutical industry will be GWA studies of drug response that identify patient stratification markers for clinical trials and guide drug improvement, particularly for avoidance of adverse events.

Despite the current euphoria, GWA studies are likely to have significant limitations. Insufficient numbers of cases will be available for GWA of uncommon traits or diseases. Current GWA genotyping methods will not identify associations with rare variants, even with large effect sizes. Approximately twenty percent of the genome represents recombinational hotspots that are not amenable to LD-based approaches (International HapMap Consortium 2005). At recombinational coldspots, haplotype blocks may be too large for unambiguous identification of causal variants. The extent of the effect of copy number variation (CNV) on association signals is not yet clear. For some common traits or diseases, these considerations may reflect a substantial proportion of the genetic variance. The addition of gene expression profiling, CNV estimation and large-scale resequencing technologies to GWA studies should circumvent some of these limitations. Use of adaptive statistical methods and resampling strategies may circumvent the need for thousands of affected individuals in uncommon traits (Berry, 2004).

Clearly a huge amount of genetics, biochemistry and cell biology remains to be done to confirm the biologic relevance of associations and to elucidate the mechanisms of genotype-phenotype associations. For geneticists, a long term goal is to piece together the genetic architecture of complex traits, evaluating with much greater precision the genetic model and contributions of factors such as epistasis, genocopies, phenocopies and penetrance.

## Figures and Tables

**Table 1:** Discovery and validation designs of recent GWA studies.

Disease	Discovery Phase			Validation Phase			Reference
	Cohort size	# SNPs	Population	Cohort size	# SNPs validated/tested	Population	
AMD	146	105,980	Caucasian	96	5/5	Same	Klein et al. 2005
Bipolar Disorder	1024, pooled	555,235	Western European	1648	88/1877	Same	Baum et al. 2007
Breast Cancer	754	227,876	European	45,426	7/30	same	Easton et al. 2007
Breast Cancer	2287	528,173	European	3848	2/8	same	Hunter et al. 2007
Breast Cancer	13,163	311,524	Icelandic	7968	2/9	various	Stacey et al. 2007
Crohn’s disease	1923	304,413	European	2150	8/20	Same	Rioux et al. 2007
Crohn’s disease	1103	16,360 Non-synonymous	German	2670	3/72	Same	Hampe et al. 2007
Crohn’s disease	1475	317,497	Belgian	2236	10/10	Same	Libioulle et al. 2007
IBD	1095 + 834	308,332	European	2885	10/27	Same	Duerr et al. 2006
Late-Onset	1086	502,627	Caucasian	N.D.	—	—	Coon et al. 2007
Alzheimer’s Memory	341	502,627	Swiss	680	1/2	Several	Papassotiropoulos et al. 2006
Myocardial Infarction	752	65,671 cSNPs	Japanese	2137	4/26	same	Ozaki et al. 202
Obesity	4862	490,032	British/Irish	29,596	1/1	same	Frayling et al. 2007
Prostate Cancer	4517	316,515 & 243,957 haplotypes	Icelandic	3655	2/2	various	Gudmundsson et al. 2007
Prostate Cancer	2339	550,000	European	6266	2/2		Yeager et al. 2007
T2DM	2335	315,635	Finnish	2473	10/80	same	Scott et al. 2007
T2DM	16,179	393,453	European	9103	10/77	same	Zeggini et al. 2007
T2DM	7805	313,179 & 339,846 haplotypes	Icelandic/Danish	3382	2/47	same	Steinthorsdottir et al. 2007
T2DM	1316	392,935	French	5511	8/57	same	Sladek et al. 2007
T2DM & Triglyeride levels	2931	386,731 & 284,968 haplotypes	Finnish/Swedish	10,850	3/107	several	Diabetes Genetics Initiative, Broad Inst. et al. 2007

**Abbreviations**: AMD, age-related macular degeneration; IBD, inflammatory bowel disease; T2DM, type II diabetes mellitus; ND, not determined.

**Table 2:** Most significant associated Haplotype Blocks (HB) in GWA studies.

Disease	Most significant HB	OR of Most significant HB	Cumulative OR	# Alleles in cumulative OR	Reference
AMD	CFH	7.4	N.D.	—	Klein et al. 2005
Bipolar disorder	DGKH	1.59	3.8	19	Baum et al. 2007
Breast Cancer	FGFR2	1.26	3.6% of variance	5	Easton et al. 2007
Breast Cancer	FGFR2	1.2	N.D.	—	Hunter et al. 2007
Breast Cancer	BC029912	1.28	PAR 25%	2	Stacey et al. 2007
Crohn’s disease	ATG16L1	1.45	N.D.	—	Rioux et al. 2007
Crohn’s disease	ATG16L1	1.45	1.45	28	Hampe et al. 2007
Crohn’s disease	IL23R	2.92	N.D.	—	Libioulle et al. 2007
IBD	IL23R	1.56/0.26	N.D.	—	Duerr et al. 2006
Late-Onset	APOE ∈ 4	4.01	N.D.	—	Coon et al. 2007
Alzheimer’s Memory	KIBRA	1.24	N.D.	—	Papassotiropoulos et al. 2006
Myocardial Infarction	LTA	1.69	N.D.	—	Ozaki et al. 2002
Obesity	FTO	1.31	N.D.	—	Frayling et al. 2007
Prostate Cancer	8q24, locus 1	1.71	PAR 13%	2	Gudmundsson et al. 2007
Prostate Cancer	8q24, locus 2	1.26			Yeager et al. 2007
T2DM	TCF7L2	1.37	~20% incidence	10	Scott et al. 2007
T2DM	TCF7L2	1.37	N.D.	—	Zeggini et al. 2007
T2DM	CDKAL1	1.20	N.D.	—	Steinthorsdottir et al. 2007
T2DM	SLC30A8	1.65 heterozygous relative risk	PAR 70%	5	Sladek et al. 2007
T2DM	KCNJ11, PPARG, TCF7L2	1.14–1.48	5.71	6	WTCCC 2007
T2DM & Triglyeride levels	Non-coding near CDKN2A/B	1.20	2.3% of variance	8	Diabetes Genetics Initiative, Broad Inst. et. al. 2007

**Abbreviations**: PAR, Population attributable risk; AMD, age-related macular degeneration; IBD, inflammatory bowel disease; T2DM, type II diabetes mellitus; HB, haplotype block; OR, odds ratio; ND, not determined.

**Table 3:** Candidate variant and functional consequence in associated Haplotype Block (HB).

Disease	Most significant HB	Variant type	HB allele frequency	Functional consequence	Reference
AMD	CFH exon 9	Non-synonymous	0.38	Complement dysregulation	Klein et al. 2005
Bipolar disorder	DGKH, introns 1 & 7	Various	0.22–0.44	Not known	Baum et al. 2007
Breast Cancer	FGFR2, intron 2	Non-coding	0.48	Unknown	Easton et al. 2007
Breast Cancer	FGFR2, intron 2	Non-coding	0.49	Unknown	Hunter et al. 2007
Breast Cancer	BC029912	Synonymous	0.50	Unknown	Stacey et al. 2007
Crohn’s Disase	IL23R	Non-synonymous	0.07	As for IL23R above	Libioulle et al. 2007
Crohn’s Disease	ATG16L1	Non-synonymous	0.47	Altered autophagy	Hampe et al. 2007
IBD	IL23R exons 5–11, 3′ intergenic region	Non-synonymous, non coding	0.07	T cell mediated inflammation	Duerr et al. 2006
Late-Onset	APOE ∈4	Non-synonymous	0.14	Unknown	Coon et al. 2007
Alzheimer’s Memory	KIBRA, intron 9	Non-coding	0.48	Unknown	Papassotiropoulos et al. 2006
Myocardial infarction	BAT - LTA	Various	N.K.	Not known	Ozaki et al. 2002
Obesity	FTO, intron 1 & 2, exon 2	Various	0.41	Unknown	Frayling et al. 2007
Prostate Cancer	8q24, 300kb	Unknown	Various	Unknown	Gudmundsson et al. 2007 & Yeager et al. 2007
T2DM	TCF7L2	Intronic	0.28	Transcriptional control of insulin synthesis	Scott et al. 2007 & Zeggini et al. 2007
T2DM	CDKAL1, intron 5	N.K. (LD block 202 kb)	0.5	unknown	Steinthorsdottir et al. 2007
T2DM	SLC30A8	Non-synonymous	0.38	Insulin synthesis	Sladek et al. 2007
T2DM & triglyceride levels	CDKN2A/B	Non-coding	0.36	unknown	Diabetes Genetics Initiative, Broad Inst. et al. 2007

**Abbreviations**: AMD, age-related macular degernation; IBD, inflammatory bowel disease; T2DM, type II diabetes mellitus; HB, haplotype block; LD, linkage disequilibrium; NK, not known.
